# Dietary flaxseed oil and vitamin E improve semen quality *via* propionic acid metabolism

**DOI:** 10.3389/fendo.2023.1139725

**Published:** 2023-04-14

**Authors:** Chongshan Yuan, Kaiyan Zhang, Zhe Wang, Xin Ma, Hongyu Liu, Jing Zhao, Wenfa Lu, Jun Wang

**Affiliations:** ^1^ Joint Laboratory of the Modern Agricultural Technology International Cooperation, Ministry of Education, Jilin Agricultural University, Changchun, Jilin, China; ^2^ Key Lab of the Animal Production, Product Quality, and Security, Ministry of Education, Jilin Agricultural University, Changchun, Jilin, China

**Keywords:** cryopreservation, bull semen, flaxseed oil, vitamin E, metabolomics, transcriptomics

## Abstract

**Introduction:**

Flaxseed oil (FO) and vitamin E (VE) both have antioxidant effects on sperm. The present study investigated the effects of dietary supplementation with FO and/or VE on semen quality.

**Methods:**

16 fertile Simmental bulls were selected and randomly divided into 4 groups (n = 4): the control group (control diet), FO group (control diet containing 24 g/kg FO), VE group (control diet containing 150 mg/kg VE) and FOVE group (control diet containing 150 mg/kg VE and 24 g/kg FO), and the trial lasted 10 weeks.

**Results:**

The results showed that the addition of FO independently can increase sperm motion parameters, the levels of catalase (CAT), glutathione peroxidase (GSH-Px), testosterone (T) and estradiol (E2), while reduce oxidative stress in seminal plasma (P < 0.05). Supplement of VE independently can increased the motility, motility parameters, CAT and superoxide dismutase (SOD) levels, and reduce oxidative stress in seminal plasma (P < 0.05). There was an interaction effect of FO × VE on motility and reactive oxygen species (ROS), while GSH-Px and ROS were affected by week × VE 2-way interaction, levels of T and E2 were also affected by the dietary FO × week interaction (P < 0.05). The triple interaction effects of FO, VE and week were significant for malondialdehyde (MDA) (P < 0.05). Compared with the control group, sperm from the FOVE group had a significantly higher in vitro fertilization (IVF) rate, and subsequent embryos had increased developmental ability with reduced ROS levels at the eight-cell stage, then increased adenosine triphosphate (ATP) content and gene expression levels of CAT, CDX2, Nanog, and SOD at the blastocyst stage (P < 0.05). Metabolomic and transcriptomic results indicated that dietary supplementation of FO and VE increased the expression of the metabolite aconitic acid, as well as the expression of ABAT and AHDHA genes.

**Conclusion:**

With in-silico analysis, it can be concluded that the effects of dietary FO and VE on improving semen quality and embryo development may be related to increased aconitic acid via the ABAT and AHDHA genes involved in the propionic acid metabolism pathway.

## 1 Introduction

Cryopreservation is the most practical approach for long-term storage of bull sperm. However, most sperm are damaged by oxidative stress during cryopreservation. This damage causes a loss and decline in motility, viability and fertilization ability. Therefore, improving the antioxidant capacity of frozen-thawed sperm and reducing the level of oxidative stress is particularly important for maintaining semen quality (1).

ω-3 Polyunsaturated fatty acids (ω-3 PUFAs) play important roles in the regulation of reproductive processes such as testosterone synthesis, sperm acrosome integrity and membrane fluidity (2). Ruminants lack the relevant fatty acid desaturase enzymes and are not able to synthesize ω-3 PUFAs. Thus, these animals must obtain ω-3 PUFAs or their pre-cursors from dietary sources (3). Flaxseed oil (FO) contains up to 50% ω-3 PUFAs, of which α-linolenic acid is converted to eicosapentaenoic and docosahexaenoic acids in mammals (4). In addition, ω-3 PUFAs contain stearic, oleic, linoleic and palmitic acids, and phenolic compounds, all of which have important roles in spermatogenesis and antioxidant enzyme activity (5).

Vitamin E (VE), one of the major antioxidants against reactive oxygen species (ROS) and lipid peroxidation, has been shown to be a major component of the antioxidant system of sperm (6, 7). VE deficiency may lead to developmental arrest of reproductive organs, including testicular tissue damage, and reduction of spermatogonia and spermatocytes (8). Previous studies demonstrated that supplementation of diets with VE could improve semen quality by reducing mitochondrial protection from oxidative stress damage (9).

In the past decade, *in vitro* fertilization (IVF) technology has made significant progress in animal reproduction (10). Compared with artificial insemination, IVF is more cost-effective and less time-consuming (11). Seminal plasma metabolomics has been used to screen for potential biomarkers of fertility in bulls (12). To investigate male fertility, transcriptome sequencing technology has been used to assess mRNA expression in sperm (13, 14). Therefore, metabolomic and transcriptomic analyses are important tools for identifying semen fertility.

At present, there is no relevant report on the effect of the combination of FO and VE in the diet on the frozen-thawed semen of bulls. Therefore, we speculate that the combined addition of FO and VE in the diets could further reduce oxidative stress and improve motility and motion parameters of frozen-thawed sperm, compared with FO or VE alone. At the same time, the combined addition of FO and VE could further improve the ability of IVF and early embryonic development by enhancing the antioxidant capacity. The aim of this study was to evaluate the effects of combined FO and VE supplementation on bull semen quality and subsequent embryonic development *in vitro*, and to explore the mechanism of the effects of FO and VE on semen quality using transcriptome and metabolome analyses.

## 2 Materials and methods

### 2.1 Experimental design

The trial lasted for 10 weeks, and 16 fertile Simmental bulls (approximately 2–3 years of age and 550–650 kg each) were selected from Changchun Xinmu Sciences & Technologies Co., Ltd, and divided randomly into four groups (n = 4). They were all kept under similar management. Ingredients and chemical composition of the diets are shown in **Table 1**. The dietary treatments were as follows: 1) control group: control diet, 2) FO group: control diet containing 24 g/kg FO (15), 3) VE group: control diet containing 150 mg/kg VE (16), and 4) FOVE group: control diet containing 150 mg/kg VE and 24 g/kg FO (17). In the current study, FO was purchased from Inner Mongolia Jiuding Food Co., Ltd., China. The molar percentages of linolenic acid, linoleic acid, oleic acid and saturated acyl groups in FO was 55.7 ± 0.0%, 14.2 ± 0.3%, 20.5 ± 1.2% and 9.5 ± 0.9% respectively (18). VE was purchased from Zhejiang NHU Co., Ltd., China. This study showed that the combination of FO and VE improved the motion parameters of sperm and the antioxidant capacity of seminal plasma after frozen-thawed at week 10. Therefore, the control and FOVE groups of week 10 samples were selected for subsequent IVF, seminal plasma metabolome and sperm transcriptome studies.

**Table 1:** Ingredients and chemical composition of diets.

Ingredient	% of dry matter	Chemical composition	% of dry matter
corn	45	Crude protein	20
Soybean	32	Crude ash	7
Wheat bran	5	Ca	0.7
Rice bran	5	P	0.7
Soy Germ Powder	6	NaCl	1
Molasses	2		
Premix	5		

The premix contains vitamin A (5×10^5^IU/kg), vitamin D (2×10^5^IU/kg), Cu (250mg/kg), Fe (1250mg/kg), Zn (1500mg/kg) and Mn (1000mg/kg).

### 2.2 Semen collection and assessment

Semen was collected by experienced technicians, twice a week per bull, into a heated (38°C) artificial vagina connected to a disposable tube, and quickly transferred to an adjacent laboratory where the physiological parameters were assessed after semen collection. Semen samples were expanded in a BioXcell^®^ (IMV Technologies, L’agile, France) extender to a concentration of 1.28 × 10^8^, then loaded into thin tubes (IMV Technologies), and the semen was equilibrated at 4°C for 2 h, and subsequently cooled at approximately 8 min from 4°C to − 140°C by a turbofreezer (Minitube, Germany) as previously described by Memon et al. (19). After that, samples were stored in a liquid nitrogen tank (−196°C). The sperm motility and motion parameters were assessed using the Hamilton Thorne IVOS II automatic sperm analyzer (Hamilton-Thorne, Beverly, MA, USA). For each bull, 2 straws were thawed by immersion in a water bath at 37°C for 30 s. 10 μL were collected from each straw, and eight fields were randomly examined.

### 2.3 Oxidative stress index and hormones

The oxidative stress index and hormones of embryo culture medium and seminal plasma included superoxide dismutase (SOD, MM-34758O1, 1.2U/mL-42 U/mL), catalase (CAT, MM-50463O1, 0.5 U/mL-18U/mL), glutathione peroxidase (GSH-Px, MM-2465O1, 20 IU/L-480 IU/L), ROS (MM-50460O1, 5 IU/mL-160 IU/mL), malondialdehyde (MDA, MM-34745O1, 0.5 nmol/mL-16 nmol/mL), testosterone (T, MM-2382O1, 0.3nmol/L-12nmol/L) and estradiol (E_2_, MM-0023O1, 4 pmol/L-120 pmol/L) were determined using specific enzyme-linked immunoassay kits (Jiangsu Meimian Industrial Co., Ltd, Yancheng, China), according to the manufacturer’s instructions. Briefly, semen was centrifuged at 3000 rpm for 15 min, and the seminal plasma was extracted before analysis. Results were measured at 450 nm on a spectrophotometer (Shanghai Spectrophotometer Co., Ltd, Shanghai, China).

### 2.4 *In vitro* maturation

Oocyte collection and IVM were performed as described previously (20). Briefly, a 10 mL syringe was used to aspirate cumulus–oocyte complexes from antral follicles with a diameter between 2 and 8 mm. Oocytes surrounded by at least three cumulus cell layers were selected for subsequent experiments, then washed in collection medium M199 supplemented with 2% fetal bovine serum (FBS, Hyclone) and transferred to maturation medium modified M199 medium supplemented with alanyl-glutamine (10 μL/mL), sodium pyruvate (50 μL/mL), epidermal growth factor (10 μL/mL), follicle-stimulating hormone (4 μL/mL), luteinizing hormone (4 μL/mL), E_2_ (10 μL/mL), cysteamine (10 μL/mL), and 10% FBS, incubated for 22 h at 38.5°C, 5% CO_2_ in air and high humidity.

### 2.5 *In vitro* fertilization

After 22 h, cumulus–oocyte complexes were added to SOF-IVF medium (6.29 mg/mL NaCl, 0.53 mg/mL KCl, 0.16 mg/mL NaH_2_PO_4_, 0.1 mg/mL MgCl_2_·6H_2_O, 0.25 mg/mL CaCl_2_·6H_2_O, 0.758 mg/mL sodium lactate, 2.1 mg/mL NaHCO_3_, 6.25 mg/mL BSA, 10.417 μL/mL MEM non-essential amino acids, 10 μL/mL fructose, 10 μL/mL heparin and 10 μL/mL sodium pyruvate). Frozen-thawed pooled semen was washed through 45% and 90% Percoll gradients (21). Semen, layered on the top, was centrifuged (700 ×g, 15 min). 5 mL of sperm TALP medium (5.84 mg/mL NaCl, 0.23 mg/mL KCl, 35 μg/mL NaH_2_PO_4_, 2.1 mg/mL NaHCO_3_, 2.38 mg/mL HEPES, 80 μg/mL MgCl_2_·6H_2_O, 310 μg/mL CaCl_2_·6H_2_O, 3.07 μL/mL sodium lactate, 6 mg/mL BSA and 0.11 mg/mL sodium pyruvate) were used to wash the pellet (300 ×g, 5 min). Sperm concentration was calculated with a hemocytometer and adjusted to 10^6^/mL with SOF-IVF medium.

### 2.6 *In vitro* culture

After 18 h of co-incubating cumulus–oocyte complexes and sperm at 38.5°C, 5% CO_2_ in air and high humidity, Zygotes were mechanically separated *via* pipetting and washed three times through IVC medium SOF-aa with 62.94 mg/mL NaCl, 5.33 mg/mL KCl, 1.62 mg/mL NaH_2_PO_4_, 0.46 mg/mL MgCl_2_, 4.7 mg/mL sodium lactate, 4.32 mg/mL glucose, 26.07 mg/mL NaHCO_3_, 2.52 mg/mL CaCl_2_·2H_2_O, 0.36 mg/mL sodium pyruvate, 20 μL/mL essential amino acids, 10 μL/mL non-essential amino acids, 4 mg/mL BSA and 0.15 mg/mL glutamine. The zygotes were cultured at 38.5°C, 5% CO_2_ in air and high humidity. The number of two-, four- and eight-cell embryos were recorded at 32, 42 and 72 h, respectively, and blastocyst yield was assessed at Day 7.

### 2.7 Measurement of intracellular ROS levels

Intracellular ROS levels in embryos were measured with a ROS detection kit (S0033S; Beyotime, China). Briefly, embryos were incubated with 10 mM DCFH-DA for 20 min at 37°C. After washing three times with 1% phosphate-buffered saline/PVP360 (PBS-PVP), the embryos were examined using a fluorescence microscope (Olympus, Tokyo, Japan) with a 460 nm excitation filter. Fluorescence images of all embryos were recorded using a digital camera (Nikon 990, Tokyo, Japan), and analyzed using the NIH ImageJ by Wayne Rasband from the National Institute of Health (Bethesda, MD, USA). Fluorescence intensities of the embryos were compared with that of the control after deducting the background value.

### 2.8 Measurement of intracellular ATP levels

Adenosine triphosphate (ATP) of embryos was detected using an ATP assay kit (S0063, Invitrogen). Briefly, embryos were collected, washed twice in PBS-PVP and then fixed in formaldehyde-PVP for 0.5 h at room temperature. Afterwards, the embryos were washed three times with PBS-PVP, 5 min each time, and then incubated with PBS-PVA containing 0.1% Triton X-100 for 1 h for membrane permeabilization. After thoroughly washing with PBS-PVP, the fluorescence signal was captured using an epifluorescence microscope, and the fluorescence intensity of embryos was analyzed using NIH ImageJ software.

### 2.9 RNA isolation, cDNA synthesis and q-PCR

Total RNA from bovine blastocysts was extracted using the RNeasy Mini kit (KIT0204, Qiagen, Hilden, Germany). A total of 18 blastocysts were used to extract RNA. The One-Step gDNA Removal and cDNA Synthesis SuperMix (AT311-03, TransGen Biotech, Beijing, China) was used to synthesize cDNA. The real-time PCR mix (25 μL) consisted of 2 μL of cDNA, 12.5 μL of SYBR green master mix, 9.5 μL of RNase-free water, and 0.5 μL each of primers (10 pmol). The primers used for the octamer-binding transcription factor 4 (*OCT-4*), caudal type homeobox 2 (*CDX-2*), *Nanog*, *SOD*, *CAT*, B-cell lymphoma 2 (*Bcl2*), *BCL2* Associated X (*Bax*) genes and 18s rRNA are listed in **Table 2**. The program used for the amplification consisted of a denaturing cycle of 3 min at 95°C, 40 cycles of PCR (95°C for 10 s, 55°C for 45 s, and 95°C for 1 min), a melting curve analysis consisting of 95°C for 1 min followed by 55°C for 1 min, and a step cycle starting at 55°C for 10 s with a 0.5°C/s transition rate, and cooling at 4°C. Relative gene expression data were analyzed using q-PCR and the 2^−△△CT^ method.

**Table 2:** Primer sequences for real-time q-PCR.

Gene	Primers	Length (bp)
*18s rRNA-F*	TTGATCTTCATTGTGCTGGGTG	189
*18s rRNA-R*	CTTCCTGGGCATGGAATCCT	
*Oct4-F*	CCACCCTGCAGCAAATTAGC	184
*Oct4-R*	CCACACTCGGACCACGTCTT	
*Nanog-F*	ATAATGGTTTTGGTGAGATTGGTAG	161
*Nanog-R*	ATAAAACTCAACCATACTTAACCCC	
*CDX2-F*	AAGACAAATACCGGGTCGTG	154
*CDX2-R*	CTGCGGTTCTGAAACCAAAT	
*CAT-F*	TGGGACCCAACTATCTCCAG	178
*CAT-R*	AAGTGGGTCCTGTGTTCCAG	
*SOD-F*	AGAGGCATGTTGGAGACCTG	189
*SOD-R*	CAGCGTTGCCAGTCTTTGTA	
*Bax-F*	CGAGTTGATCAGGACCATCAT	168
*Bax-R*	ATGTGGGTGTCCCAAAGTAG	
*Bcl-2-F*	TGGATGACCGAGTACCTGAA	124
*Bcl-2-R*	GAGACAGCCAGGAGAAATCAAA	

### 2.10 Metabolite extraction and analysis

Approximately 100 μL of each seminal plasma sample was transferred to a new 1.5 mL Eppendorf tube. Then an equal volume of each sample was sampled and mixed into 100 μL of a quality control (QC) sample. Then, 300 μL of cold methanol was added, shaken and mixed, and placed at −20°C for 2 h. After centrifugation at 25,000 ×g for 10 min at 4°C, 350 μL supernatant was placed in a new Eppendorf tube, and the centrifugation step was repeated. Then 25 μL supernatant was added to 225 μL 50% methanol, and 50 μL of supernatant from each sample was taken and mixed with a QC sample, and the remaining transferred to a new 1.5 mL Eppendorf tube for testing. An Acquity UPLC HSS T3 column (100 mm × 2.1 mm, 1.8 μm, Waters, UK) was used for reversed-phase separations. The column temperature was maintained at 40°C and the metabolites were eluted at a flow rate of 0.5 mL/min. The injection volume for each sample was 5 μL. A high-resolution tandem mass spectrometer Xevo G2 XS QTOF (Waters, UK) was used to detect small molecules eluted from the column. A quality control sample was acquired after every 10 samples to evaluate the stability of the LC-MS.

### 2.11 Data processing of metabolites

Peak extraction was performed primarily through the commercial software Progenesis QI (version 2.2). The resulting LC-MS data were inputted to the R software package for principal component analysis (PCA) and partial least squares discriminant analysis (PLS-DA) to evaluate differential ions, identify differentially expressed genes, and analyze metabolite and metabolic pathways.

### 2.12 Total RNA extraction of transcriptomics

Total RNA was extracted from sperm using the RNeasy Micro kit (KIT0204, Qiagen, Hilden, Germany) according to manufacturer’s instruction. Briefly, samples were transferred to tubes containing Buffer RL and one volume of 70% ethanol and then centrifuged. Buffer RW1, DNase I, Buffer RPE and 80% ethanol were added then samples centrifuged. High quality RNA was used for mRNA library construction.

### 2.13 mRNA library construction of transcriptomics

High-quality RNA was amplified (more than 200 pg) with oligo-dT and dNTPs to generate full-length cDNA by PCR. The Agilent 2100 bioanalyzer (Thermo Fisher Scientific, MA, USA) was used to determine the average molecular length of PCR products. The DNA products were purified by an Agencourt AMPure XP-Medium kit (Thermo Fisher Scientific, USA). Single-stranded circular DNA was formatted as the final library, which used phi29 (Thermo Fisher Scientific, MA, USA) to prepare a DNA nanosphere. The DNA nanosphere was loaded into a patterned nanoarray, with single end 50 base reads generated on a BGISEQ500 platform (BGI-Shenzhen, China).

### 2.14 Statistical analysis

All data are presented as least squares means ± SD. Each individual bull was considered an experimental unit in all statistical analysis. Data on oxidative stress index, T, E_2_ and the quality of semen were analyzed for the main effects of FO and VE. The FO, VE and time interactions used the General Linear Model procedure of the SPSS Institute (19.0) with a repeated measurement analysis. Differences among least square means were determined by the Tukey test and *P* < 0.05 was considered a significant difference. Differences in embryo development, CAT, SOD and ROS levels in embryo culture medium, and relative levels of gene expression, ROS and ATP in embryos, between the control and FOVE groups were compared by the T-test (SPSS 19.0), and *P* < 0.05 was considered a significant difference.

## 3 Results

### 3.1 Effect of dietary FO and VE on frozen-thawed sperm motility and motion parameters

The diet supplemented with VE significantly enhanced motility, straight distance (DSL), straight line velocity (VSL), curvilinear velocity (VCL) at week 8 and increased average path (DAP), DSL, curvilinear distance (DCL), velocity of the average path (VAP), VSL and VCL at week 10 compared with groups with no VE supplementation (*P* < 0.05) (**Table 3**). Sperm from bulls fed a diet containing FO had significantly higher values of DAP, DSL, DCL, VAP, VSL, VCL and amplitude of lateral head displacement (ALH) at week 10 compared with other groups (*P* < 0.05). There was an interaction effect of FO × VE on motility (*P* < 0.05).

**Table 3:** Frozen-thawed sperm motility and motion parameters.

	Control	FO	VE	FOVE	Overall
Motility, %					
0 week	41.43 ± 3.52	46.35 ± 7.38	44.95 ± 11.66	43.5 ± 6.92	44.06 ± 7.33^c^
4 week	43.05 ± 1.85	40.33 ± 14.48	40.85 ± 18.49	56.87 ± 5.93	45.27 ± 12.92^c^
8 week $	53.63 ± 5.1	48.27 ± 4.12	51.03 ± 7.53	68.27 ± 3.4	55.3 ± 9.27^b^
10 week	61 ± 2.45	59.93 ± 5.46	58.05 ± 7.49	74.28 ± 2.78	63.31 ± 7.99^a^
overall	49.78 ± 8.82	48.72 ± 10.77	48.72 ± 12.77	60.73 ± 12.95	–
DAP (μm)					
0 week	11.93 ± 2.57	13.04 ± 2.59	13.16 ± 3.87	12.47 ± 2.25	12.65 ± 2.63^c^
4 week	11.1 ± 0.96	10.52 ± 4.76	10.52 ± 4.76	12.14 ± 1.04	11.07 ± 3.15^c^
8 week	16.38 ± 4.23	12.48 ± 1.38	14.36 ± 2.64	19.82 ± 1.34	15.76 ± 3.69^b^
10 week #$	16.08 ± 1.52	17.2 ± 3.3	17.54 ± 2.91	26.15 ± 4.55	19.24 ± 5.07^a^
overall	13.87 ± 3.4	13.31 ± 3.84	13.9 ± 4.17	17.65 ± 6.44	–
DSL (μm)					
0 week	9.45 ± 2.27	9.19 ± 1.38	10.43 ± 2.89	9.53 ± 1.44	9.65 ± 1.93^c^
4 week	8.46 ± 1.07	8.45 ± 3.77	8.45 ± 3.77	9.41 ± 1.03	8.69 ± 2.51^c^
8 week $	12.86 ± 4.34	9.18 ± 1.09	11.88 ± 2.28	15.51 ± 1.43	12.36 ± 3.31^b^
10 week #$	12 ± 1.65	12.61 ± 1.73	13.85 ± 2.37	19.72 ± 3.51	14.54 ± 3.84^a^
overall	10.69 ± 3	9.86 ± 2.62	11.15 ± 3.29	13.54 ± 4.86	–
DCL (μm)					
0 week	22.6 ± 4.72	23.1 ± 8.2	24.41 ± 7.86	24.33 ± 5.22	23.61 ± 6.03^c^
4 week	21.57 ± 2.15	19.5 ± 8.97	19.5 ± 8.97	23.03 ± 1.63	20.9 ± 6^c^
8 week	31.2 ± 6.75	24.13 ± 2.68	26.25 ± 4.81	37.86 ± 1.39	29.86 ± 6.73^b^
10 week #$	31.01 ± 2.28	33.23 ± 7.58	32.48 ± 5.88	51.19 ± 9.87	36.98 ± 10.56^a^
overall	26.6 ± 6.11	24.99 ± 8.35	25.66 ± 7.94	34.1 ± 12.87	–
VAP (μm/sec)					
0 week	33.04 ± 6.15	36.22 ± 6.52	35.55 ± 12.3	35.52 ± 5.64	35.08 ± 7.37^c^
4 week	32.67 ± 2.01	29.72 ± 15.73	29.72 ± 15.73	37.88 ± 3.98	32.5 ± 10.71^c^
8 week	43.8 ± 9.11	36.97 ± 3.73	37.56 ± 6.38	56.34 ± 4.99	43.67 ± 9.86^b^
10 week #$	49.64 ± 3.55	52.39 ± 9.56	48.38 ± 8.81	74.09 ± 9.55	56.13 ± 13.1^a^
overall	39.79 ± 9.13	38.83 ± 12.37	37.8 ± 12.33	50.96 ± 17.1	–
VSL (μm/sec)					
0 week	26.25 ± 4.69	26.07 ± 3.3	28.28 ± 9.19	27.47 ± 3.57	27.02 ± 5.19^c^
4 week	25.23 ± 2.77	24.03 ± 12.7	24.03 ± 12.7	30.04 ± 3.94	25.83 ± 8.7^c^
8 week $	34.44 ± 9.11	27.73 ± 2.72	31.28 ± 5.71	44.76 ± 4.98	34.55 ± 8.52^b^
10 week #$	37.85 ± 3.41	39.49 ± 4.83	38.65 ± 6.94	56.94 ± 7.39	43.23 ± 9.73^a^
overall	30.94 ± 7.44	29.33 ± 8.9	30.56 ± 9.78	39.8 ± 13.13	–
VCL (μm/sec)					
0 week	61.23 ± 12.05	69.09 ± 14.7	64.59 ± 24.03	67.41 ± 13.04	65.58 ± 15.21^c^
4 week	61.7 ± 4.69	53.77 ± 28.76	53.77 ± 28.76	69.88 ± 7.06	59.78 ± 19.82^c^
8 week $	82.05 ± 15.17	69.25 ± 6.99	67.41 ± 11.66	104.88 ± 6.23	80.9 ± 18.14^b^
10 week #$	93 ± 7.07	98.21 ± 21.53	87.78 ± 17.44	140.84 ± 21.6	104.96 ± 27^a^
overall	74.5 ± 16.93	72.58 ± 24.23	68.39 ± 23.03	95.75 ± 33.2	–
ALH (μm)					
0 week	3.45 ± 0.56	4.06 ± 0.97	3.57 ± 1.32	3.89 ± 0.76	3.74 ± 0.88^c^
4 week	3.78 ± 0.32	3.02 ± 1.56	3.02 ± 1.56	4.22 ± 0.28	3.51 ± 1.14^c^
8 week	4.82 ± 0.62	4.26 ± 0.5	3.78 ± 0.49	6.06 ± 0.28	4.73 ± 0.98^b^
10 week #	5.52 ± 0.42	5.55 ± 1.26	4.88 ± 1	7.54 ± 0.96	5.87 ± 1.34^a^
overall	4.39 ± 0.96	4.22 ± 1.38	3.81 ± 1.25	5.43 ± 1.63	–

Data shown are the mean ± SD (n = 4 replicates per treatment, one ejaculate per replicate). For each parameter, values within columns with different superscript letters differ significantly (P < 0.05). #Significant (P < 0.05) main effect of flaxseed oil (FO). $Significant (P < 0.05) main effect of vitamin E (VE). CASA, computer-aided sperm analysis; Means (± SD) of the distance of the average path (DAP, μm), straight distance (DSL, μm), curvilinear distance (DCL, μm), velocity of the average path (VAP, μm/sec), straight line velocity (VSL, μm/sec), curvilinear velocity (VCL, μm/sec), amplitude of lateral head displacement (ALH, μm) were assessed after thawing. "-" This symbol has no meaning.

### 3.2 Effect of dietary FO and VE on frozen-thawed semen plasma antioxidant status and hormones

As shown in **Table 4**, dietary FO significantly increased GSH-Px and decreased MDA at week 10. Supplementation with VE significantly increased SOD and decreased MDA at week 8 compared with groups with no VE supplementation (*P* < 0.05). The diet supplemented with FO or VE alone significantly increased CAT and decreased ROS at weeks 8 and week 10 compared with no FO or VE supplementation (*P* < 0.05). There was an effect of week × VE interaction on GSH-Px and ROS, and ROS was also affected by a dietary FO × VE 2-way interaction (*P* < 0.05). The triple interaction effects of FO, VE and week were significant for MDA (*P* < 0.05). Levels of T and E_2_ in frozen-thawed semen plasma are also shown in **Table 4**. Samples from bulls fed the diet with FO had increased levels of T and E_2_ at weeks 4, 8 and 10 compared with those from bulls fed the diet without FO. At week 8, elevated T content was observed with supplementation of VE in the diet (*P* < 0.05). Levels of T and E_2_ were also affected by the dietary FO × week interaction (*P* < 0.05).

**Table 4:** Frozen-thawed semen plasma antioxidant status and hormones.

	Control	FO	VE	FOVE	overall
CAT					
0 week	187.67 ± 31.81	194.32 ± 20.57	192.65 ± 30.21	182.69 ± 25.16	189.33 ± 24.86^b^
4 week	181.03 ± 46.79	174.38 ± 23.26	177.7 ± 23.26	192.65 ± 28.71	181.44 ± 29.49^b^
8 week #$	187.67 ± 9.97	240.83 ± 19.84	249.13 ± 27.4	252.46 ± 12.13	232.52 ± 31.82^a^
10 week #$	139.5 ± 19.56	177.7 ± 23.88	182.69 ± 8.58	200.96 ± 9.97	175.21 ± 27.54^b^
overall	173.97 ± 34.16	196.81 ± 33.7	200.55 ± 36.41	207.19 ± 33.38	–
GSH-Px					
0 week	431.54 ± 32.9	448.73 ± 36.51	402.24 ± 44.73	451.6 ± 16.13	433.53 ± 36.69^b^
4 week	420.39 ± 27.17	461.15 ± 41.98	466.88 ± 68.15	479.62 ± 30.69	457.01 ± 46.28^b^
8 week	577.39 ± 43.67	611.47 ± 27.84	543.95 ± 51.65	589.18 ± 51.59	580.5 ± 47.3^a^
10 week #	339.82 ± 15.05	377.08 ± 64.77	430.58 ± 17.85	467.2 ± 24.38	403.67 ± 60.1^b^
overall	442.28 ± 92.83	474.61 ± 96.81	460.91 ± 70.27	496.9 ± 63.44	–
SOD					
0 week	42.78 ± 5.13	43.15 ± 1.61	42.44 ± 2.1	40.88 ± 8.73	42.31 ± 4.76^b^
4 week	44.04 ± 2.68	44.86 ± 4.5	45.08 ± 3.52	49.35 ± 2.27	45.83 ± 3.68^b^
8 week $	49.98 ± 7.22	57.74 ± 6.43	58.74 ± 8.98	58.89 ± 3.04	56.34 ± 7.16^a^
10 week	38.99 ± 4.91	44.37 ± 3.03	41.7 ± 2.29	44.56 ± 1.7	42.41 ± 3.72^b^
overall	43.95 ± 6.21	47.53 ± 7.22	46.99 ± 8.44	48.42 ± 8.2	–
MDA					
0 week	12.71 ± 3.92	13.95 ± 3.87	14.15 ± 2.8	13.99 ± 2.18	13.7 ± 2.99b
4 week	14.41 ± 1.52	13.36 ± 1.56	13.78 ± 0.68	10.05 ± 3.12	12.9 ± 2.45b
8 week $	17.51 ± 3.83	17.21 ± 2.44	16.71 ± 1.9	13.04 ± 1.11	16.12 ± 2.92a
10 week #	13.45 ± 2.62	7.55 ± 0.79	8.79 ± 0.89	8.92 ± 2.21	9.68 ± 2.83c
overall	14.52 ± 3.38	13.02 ± 4.21	13.36 ± 3.36	11.5 ± 2.96	–
ROS					
0 week	126.86 ± 25.94	130.56 ± 13.73	129.82 ± 27.3	131.02 ± 12.53	129.56 ± 18.86^b^
4 week	123.08 ± 16.84	127.23 ± 9.42	139.05 ± 11.17	146.81 ± 12.85	134.04 ± 15.07^b^
8 week #$	204.72 ± 21.8	176.92 ± 19.98	159.83 ± 11.32	156.97 ± 8.05	174.61 ± 24.45^a^
10 week #$	131.39 ± 4.4	93.34 ± 6.34	78.65 ± 11.04	85.58 ± 4.21	97.24 ± 21.98^c^
overall	146.51 ± 38.78	132.01 ± 32.96	126.84 ± 34.29	130.1 ± 29.61	–
T (nmol/L)					
0 week	15.28 ± 1.46	13.99 ± 1.22	14.54 ± 0.55	15.28 ± 1.14	14.77 ± 1.16^b^
4 week #	15.92 ± 0.98	17.68 ± 0.97	16.66 ± 2.01	19.61 ± 1.43	17.47 ± 1.91^b^
8 week #$	19.7 ± 0.46	22.1 ± 2.73	21.36 ± 2.12	25.33 ± 1.09	22.13 ± 2.67^a^
10 week #	9.37 ± 1.42	14.26 ± 0.6	9.93 ± 0.18	13.99 ± 0.55	11.89 ± 2.44^c^
overall	15.07 ± 3.95	17.01 ± 3.68	15.62 ± 4.45	18.55 ± 4.68	–
E_2_ (pmol/L)					
0 week	232.06 ± 28.15	212.07 ± 30.18	238.91 ± 36.85	222.62 ± 19.8	226.41 ± 28.28^c^
4 week #	226.59 ± 10.17	269.16 ± 12.26	217.82 ± 7.76	278.88 ± 18.09	248.11 ± 29.45^b^
8 week #	264.78 ± 27.96	299.42 ± 54.78	292.71 ± 19.57	317.77 ± 44.5	293.67 ± 40.19^a^
10 week #	113.64 ± 12.68	129.24 ± 22.32	116.79 ± 14.21	135.82 ± 4.78	123.87 ± 16.24^d^
overall	209.27 ± 62.02	227.48 ± 73.43	216.56 ± 68.81	238.77 ± 74.39	–

Data shown are the mean ± SD (n = 4 replicates per treatment, one ejaculate per replicate). For each parameter, values within columns with different superscript letters differ significantly (P < 0.05). #Significant (P < 0.05) main effect of flaxseed oil (FO). $Significant (P < 0.05) main effect of vitamin E (VE).

### 3.3 Effect of dietary FO and VE on thawed sperm and subsequent embryo development outcomes

**Figure 1:**
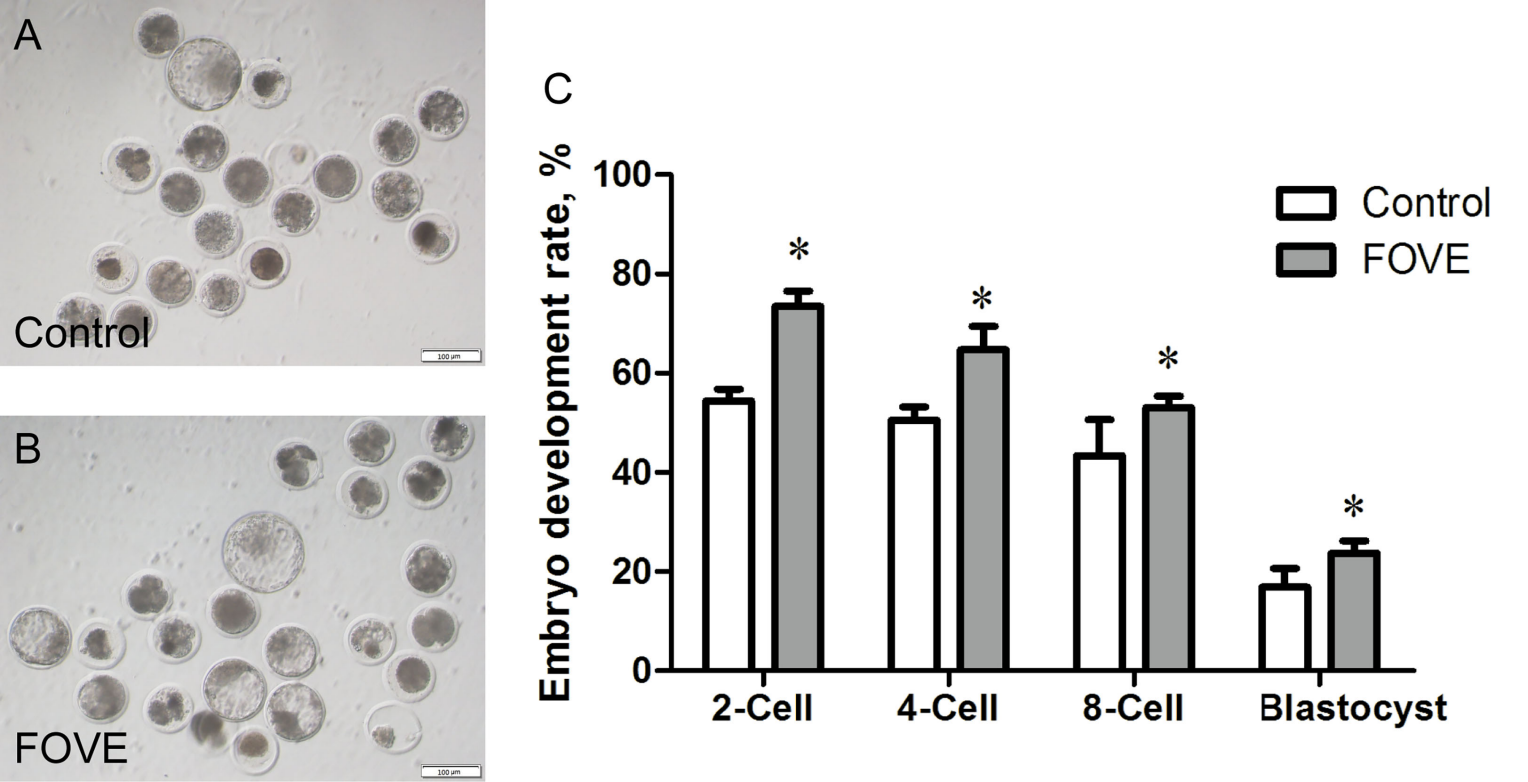
The above results show that adding FO and VE to the diet for 10 weeks can improve the motility and motion parameters of frozenthawed sperm, and also improve the antioxidant capacity, and the combined addition of FO and VE has a better effect. The control group and FOVE group samples of week 10 were used for further experiments. IVF results **(A–C)** showed that the diet supplemented with FO and VE significantly increased the twocell, four-cell, eight-cell and blastocyst rate compared with the control group (P < 0.05).

The above results show that adding FO and VE to the diet for 10 weeks can improve the motility and motion parameters of frozen-thawed sperm, and also improve the antioxidant capacity, and the combined addition of FO and VE has a better effect. The control group and FOVE group samples of week 10 were used for further experiments. IVF results (**Figure 1**) showed that the diet supplemented with FO and VE significantly increased the two-cell, four-cell, eight-cell and blastocyst rate compared with the control group (*P* < 0.05).

### 3.4 Effects of dietary FO and VE on the ROS and ATP of bovine embryos

**Figure 2:**
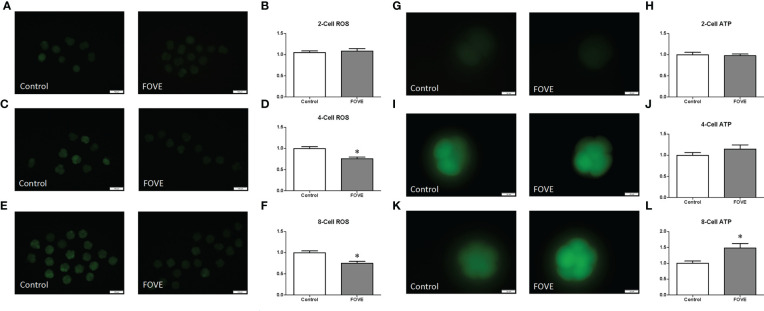
Intracellular ROS levels were measured by assessing DCFH fluorescence **(A–F)**. Quantitative analysis showed that the ROS levels of four- and eight-cell embryos were significantly decreased (P < 0.05) in the FOVE group compared with the control group **(C–F)**. There were no significant difference in ATP content between two- and four-cell shown in **(G–J)** (P > 0.05). As shown in Figures 2K, L, the ATP levels of eight-cell embryos were significantly higher in the FOVE group than in the control group (P < 0.05).

Intracellular ROS levels were measured by assessing DCFH fluorescence (**Figures 2A–F**). Quantitative analysis showed that the ROS levels of four- and eight-cell embryos were significantly decreased (*P* < 0.05) in the FOVE group compared with the control group (**Figures 2C–F**). As shown in **Figures 2K, L**, the ATP levels of eight-cell embryos were significantly higher in the FOVE group than in the control group (*P* < 0.05).

### 3.5 The effect of dietary FO and VE on the antioxidant status of embryo culture medium

**Figure 3:**
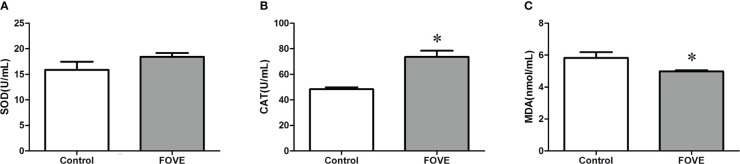
Antioxidant status of embryo culture medium. Values are shown as mean ± SD. *Significant difference compared with the control group (*P* < 0.05). **(A–C)** shows the effect of dietary FO and VE on the antioxidant status of embryo culture medium. The activity of CAT was increased when the diet was supplemented with FO and VE (P < 0.05). In contrast, FO and VE supplementation resulted in decreased MDA levels in embryo culture medium, but the activity of SOD did not differ among the groups (P > 0.05)

**Figure 3** shows the effect of dietary FO and VE on the antioxidant status of embryo culture medium. The activity of CAT was increased when the diet was supplemented with FO and VE (*P* < 0.05). In contrast, FO and VE supplementation resulted in decreased MDA levels in embryo culture medium, but the activity of SOD did not differ among the groups (*P* > 0.05).

### 3.6 The effect of dietary FO and VE on the relative levels of gene expression in blastocysts

**Figure 4:**
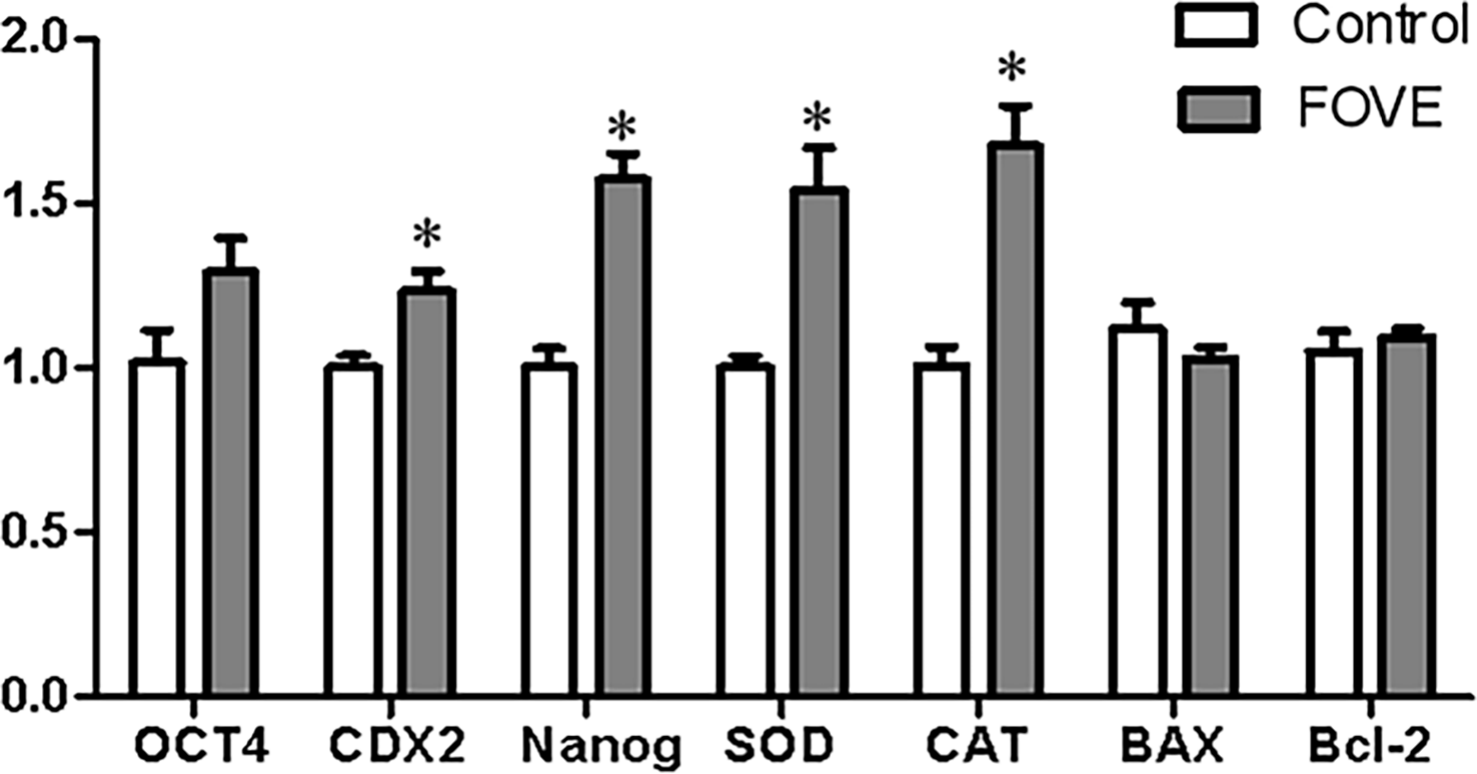
Relative levels of gene expression in blastocysts. Values are shown as mean ± SD. *Significant difference compared with the control group (*P* < 0.05).

The effects of dietary FO and VE combined on blastocyst gene expression were examined by q-PCR. The relative abundance of gene expression in blastocysts is presented in **Figure 4**. The results showed that blastocysts from the sperm of bulls consuming a diet supplemented with FO and VE had significantly increased expression of *CDX2*, *Nanog*, *SOD* and *CAT* compared with control group (*P* < 0.05).

### 3.7 Metabolomic changes and pathway enrichment analysis

**Figure 5:**
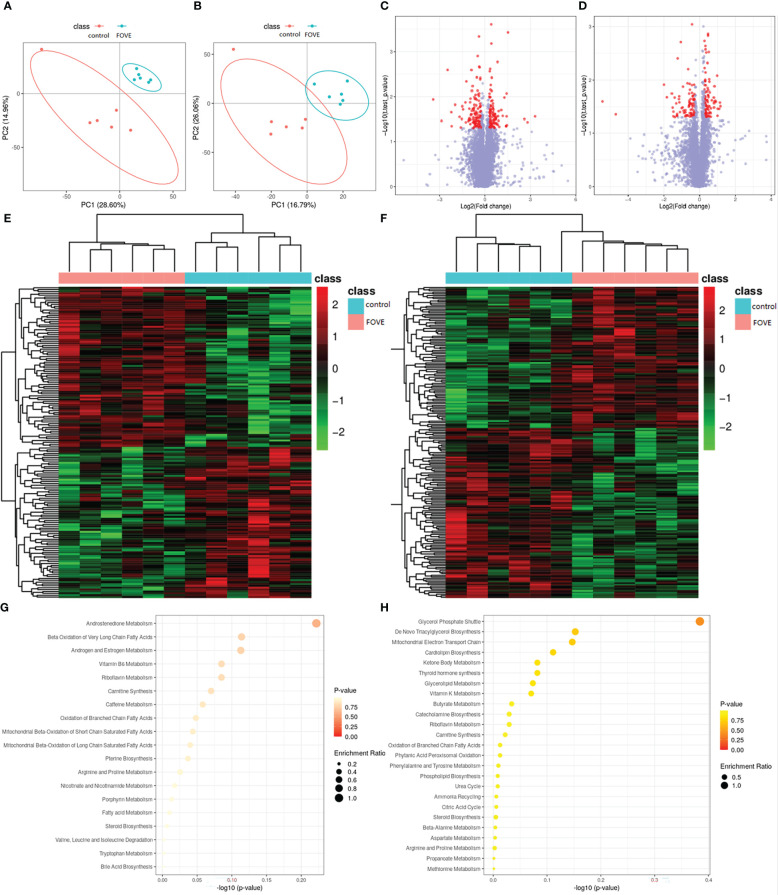
Seminal plasma metabolomic changes and pathway enrichment. Positive ion mode **(A)** and negative ion mode **(B)** for the partial least square-discriminate analysis (PLS-DA) score charts of metabolite profiling data. Analysis of the flaxseed oil + vitamin E (FOVE) group and control group based on metabolomics. Volcano figure under positive ion mode **(C)** and negative ion mode **(D)**, with the red points representing different levels of metabolites. Hierarchical clustering analysis of positive ion mode **(E)** and negative ion mode **(F)**, with red denoting increased expression and blue denoting decreased expression. The pathway analysis for positive ion mode **(G)** and negative ion mode **(H)**. Colors (varying from yellow to red) show metabolites in the data with different levels of significance. The x-axis represents the pathway impact value computed from pathway topological analysis, and the y-axis is the-log of the *P*-value obtained from pathway enrichment analysis. The pathways that were most significantly changed are characterized by both a high-log (*P*) value and high impact value (top right region).

The above experimental results suggest that the combined addition of FO and VE in the diet may improve the IVF rate of sperm and the developmental ability of early embryos by improving antioxidant capacity. Therefore, we used metabolomics and transcriptomics to deeply explore the mechanism of improving the motion parameters of frozen-thawed sperm motility and IVF ability. Significantly differences in metabolites were identified according to the PLS-DA VIP (variable importance in the projection) > 1, fold change ≥ 1.2 and *P* < 0.05 (**Figures 5A, B**). We identified a total of 12,252 positive ions (pos) and 10,101 negative ions (neg). As shown in **Figures 5C, D**, compared with the control group, 198 and 167 differentially expressed ions were identified in the pos and neg mode, respectively. Among them, 90 and 108 were up-regulated and down-regulated, respectively, in the pos mode, and 86 and 81 were up-regulated and down-regulated, respectively, in neg mode (**Figures 5E, F**). The main differences in metabolites are shown in **Table 5**. Pathway enrichment analysis was performed to study the effect of dietary FO and VE on sperm, as shown in **Figures 5G, H**. The results revealed that the metabolites with altered levels of expression were mainly associated with androgen and estrogen metabolism, riboflavin metabolism, fatty acid metabolism, steroid biosynthesis and bile acid biosynthesis in positive ion mode (**Figure 5G**), and mitochondrial electron transport chain, glycerolipid metabolism, citric acid cycle and tyrosine metabolism in negative ion mode (**Figure 5H**).

**Table 5:** Changes in the main metabolites between the control and FOVE groups.

Compared sample	Metabolite name	KEGG ID	ratio	*P*-value	VIP
Steroid biosynthesis					
FOVE: control	Lanosterol	C01724	1.454291	0.009641	2.252628
Vitamin digestion and absorption					
FOVE: control	Riboflavin	C00255	1.341744	0.0474189	1.28584909
Tryptophan metabolism;					
FOVE: control	lactate	C22006	1.243668	0.0291399	1.34673036
Cutin, suberine and wax biosynthesis					
FOVE: control	palmitic acid	C08285	1.958944	0.008367	3.23004716
Phenylalanine, tyrosine and tryptophan biosynthesis					
FOVE: control	Protocatechuic acid	C00230	1.253955	0.00267	1.884392
Propanoate metabolism					
FOVE: control	aconitate	C21250	1.214776	0.019896	1.399021

### 3.8 KEGG and Gene ontology analysis of differentially expressed genes

**Figure 6:**
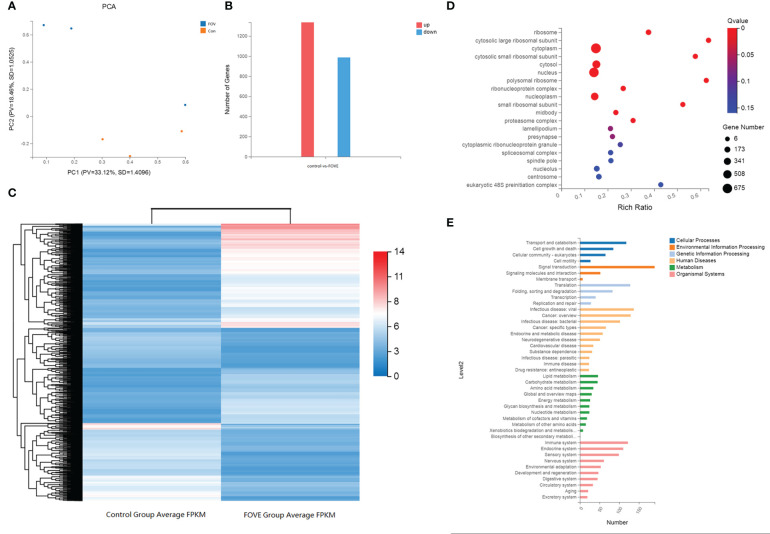
KEGG and GO analysis of differentially expressed genes. Principal component analysis (PCA) score plots of the two groups **(A)**. The number of differentially expressed genes (DEGs), red column = increased expression (n=1336) and blue column = decreased expression (n=987) **(B)**. Hierarchical clustering analysis of DEGs. Red denotes increased expression and blue denotes decreased expression **(C)**. Function analysis of differentially expressed genes (DEGs) between two treatment groups based on Gene Ontology **(D)** and KEGG pathway **(E)** analysis. D, The size of the dot designates the number of DEGs in the pathway, and the color of the dot corresponds to a different Q value (for the most enriched top 20 pathway terms). E, The ordinate is the enriched KEGG pathway term, and the abscissa is the number of DEGs for the term. Different colors are used to differentiate cellular processes, environmental information processing, genetic information processing, human diseases, metabolism and organismal systems.

The overall distribution trend of all samples was investigated by PCA, and the PCA score plots of the two groups are shown in **Figure 6A**. Variations were identified in the PC1 and PC2 score plots. Differentially expressed genes were chosen with the DESeq package using the following criteria: |log2| (fold change) > 3 and adjusted *P*-value < 0.01. The results showed that 1336 genes and 987 genes were up-regulated and down-regulated, respectively, in the FOVE group compared with the control group (**Figures 6B, C**), and the top 5 significantly up-regulated genes in the two groups are listed in **Table 6**. Gene ontology (GO) analysis of DEGs was categorized functionally based on GO annotation terms. **Figure 6D** shows the top 20 enriched GO terms. To further characterize DEGs, we performed pathway analysis using the KEGG pathway database. As shown in **Figure 6E**, the major enrichment pathways included cell motility, lipid metabolism, amino acid metabolism, nucleotide metabolism, and energy metabolism.

**Table 6:** Information of up-regulated expresse differentially genes.

Gene. ID	Gene Name	log2 Fold Change	*P*-value	KEGG pathway
100125267	*LIPA*	4.14909	2.80E-05	Steroid biosynthesis Cholesterol metabolism
280969	*ABAT*	3.820472	2.78E-10	Propanoate metabolism
281810	*HADHA*	7.471023	5.09E-41	Propanoate metabolism Fatty acid metabolism
338074	*AOX1*	8.803598209	1.27E-260	Vitamin B6 metabolism Nicotinate and nicotinamide metabolism
518852	*EHHADH*	7.202206	1.2E-167	Propanoate metabolism Fatty acid metabolism

### 3.9 Integrated analysis of the metabolomic and transcriptomic data

**Figure 7:**
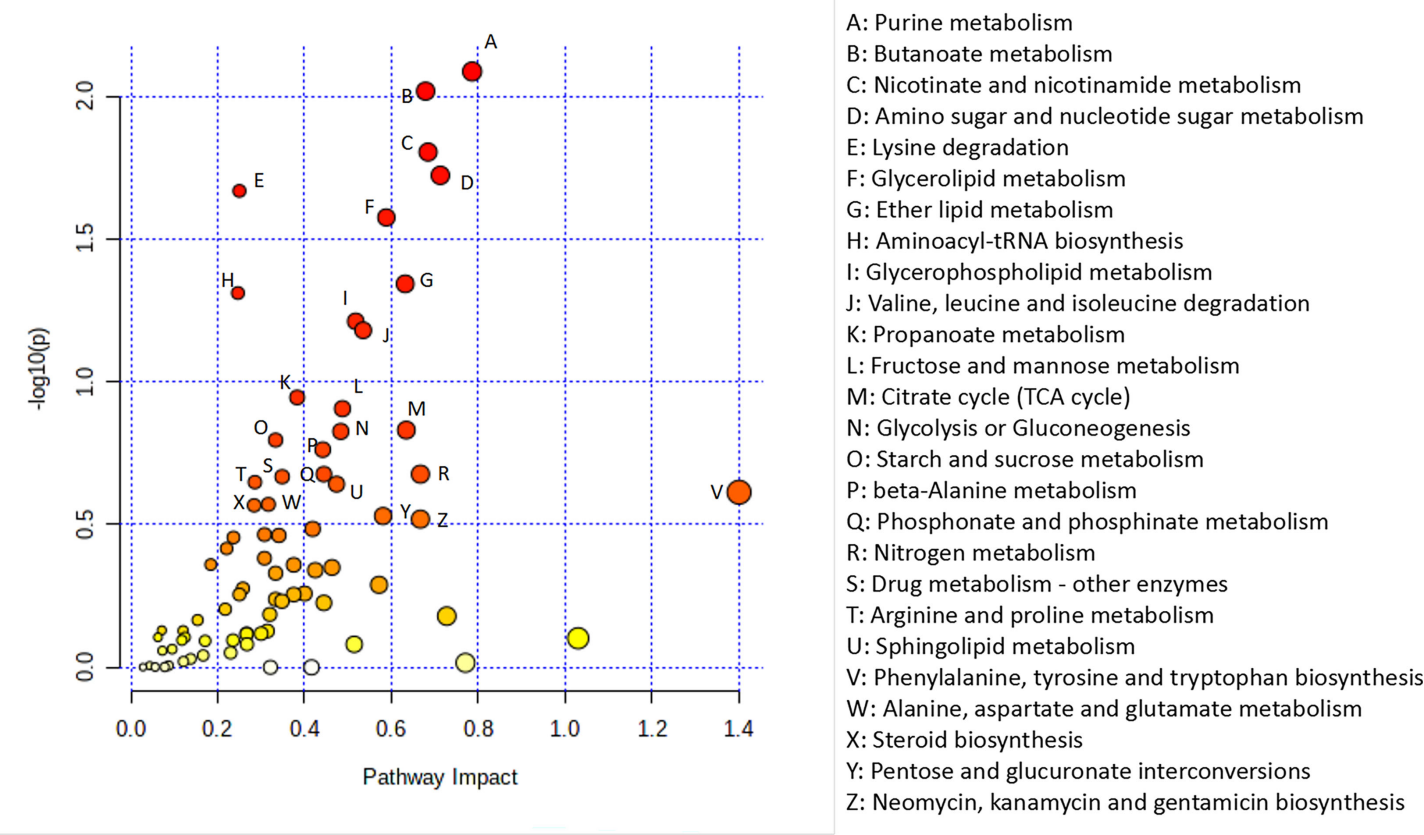
Integrated analysis of the metabolomic and transcriptomic data. Integrated analysis of the metabolomic and transcriptomic data performed with Metaboanalyst (https://www.metaboanalyst.ca/). Each point represents one metabolic pathway. The size of the dot represents a positive correlation with the metabolic pathway.

To further explore the potential relationship between DEGs and metabolites, we integrated metabolomic and transcriptomic data at the pathway level. As shown in **Figure 7**, there were a total of 77 enriched pathways, including purine metabolism, nicotinate and nicotinamide metabolism, glycerolipid metabolism, glycerophospholipid metabolism, valine, leucine and isoleucine degradation, propanoate metabolism, fructose and mannose metabolism, tricarboxylic acid cycle (TCA cycle), phenylalanine, tyrosine and tryptophan biosynthesis and steroid biosynthesis.

## 4 Discussion

In this study, metabolomics and transcriptomics were used to clarify the mechanism of dietary supplementation of FO and VE on semen quality and embryonic development. FO contains up to 50% ω-3 PUFAs, especially α-linolenic acid (22), which plays important roles in the regulation of membrane properties (23). Previous studies have shown that ω-3 PUFAs in diets improved sperm morphology in the boar (15). Dietary supplementation of VE could reduce the rate of sperm deformity in goats (9). Results of the present study indicated that dietary supplementation with 24 g/kg FO and 150 mg/kg VE had positive effects on bull sperm. The positive effects may be due to an increased level of aconitic acid *via* upregulated expression of 4-aminobutyrate aminotransferase (*ABAT*) and Hydroxyacyl-CoA dehydrogenase subunit alpha (*HADHA*) genes of the propionate metabolic pathway, providing energy for bull sperm and promoting the IVF rate and subsequent embryonic development.

Our results demonstrated that the diet supplemented with FO and VE increased frozen-thawed sperm motion parameters. These results are consistent with previous studies that showed dietary supplementation with FO alone increased the motility of sperm, progressive motility, vitality, membrane integrity and quality of frozen-thawed sperm (16, 24). Similarly, it has been shown that 8% FO in goat diets increased the antioxidant capacity of testicular tissue and promoted testicular development and spermatogenesis (17). ω-3 PUFAs can affect the hypothalamic-pituitary-gonadal axis, thereby promoting the secretion of important hormones related to reproduction, such as T and E_2_ (25, 26). Similarly, FO promoted the secretion of T and E_2_ in our study. It can be speculated that FO may promote the production of steroid hormones and then stimulate the production of sperm.

Numerous studies have shown that high concentrations of VE in the diet improved semen quantity (27, 28). As the main antioxidant of sperm, vitamin E can scavenge oxygen free radicals in sperm membrane and avoid oxidative damage to sperm (29). It has been reported that diets supplemented with VE at 200 IU/sheep/d significantly reduced MDA levels and increased the activities of SOD and GSH-Px in semen (9). Consistent with these findings, our results showed that adding VE increased frozen-thawed sperm motility and increased SOD and CAT levels and reduced MDA and ROS levels in bull seminal plasma. Taken together, it was observed in this study that VE may enhance sperm fertility through antioxidant effects (30).

It was reported that the combined addition of ω-3 PUFAs and VE may have reduced oxidation of sperm plasma membranes and maintained semen quality during cryopreservation (31). In the current study, the addition of FO and VE in the diet may have prevented sperm lipid peroxidation caused by MDA and ROS. In addition, FO and VE in the diet improved sperm motility and motion parameters, which is consistent with the results of Kargar et al., suggesting that dietary supplementation with FO and VE improved sperm motility (17). Supporting these findings, supplementation of FO and VE in the diet improved rooster semen quality and fertility potential (32), and increased the motility and membrane integrity of stallion semen (31).

Accumulating evidence has revealed that sperm motility and motion parameters are positively correlated with early embryonic development (33). Low motility sperm may affect the acrosome reaction, and the rate of fertilization and blastocyst formation (34). Sperm with low motion parameters and motility can decrease embryo cleavage rates (35). The present results suggested that dietary FO and VE improves embryo development by improving sperm motility.

During embryonic development, mitochondria provide ATP to cleavage-stage embryos by uptake and oxidation of various substrates (36). ROS is a by-product of ATP generation through oxidative phosphorylation, and cause oxidative stress and embryo damage (37). The synthesis of ROS and ATP in the cytoplasm of embryonic cells may be affected by sperm quality (38). In this study, embryos from the FOVE group showed increased ATP levels at the eight-cell stage, and decreased ROS levels at the four- and eight-cell stage, suggesting that sperm from bulls fed a diet with FO and VE may be beneficial for subsequent embryonic development.

It is reported that concentrations of ROS in spent culture media may play a critical role in the success of IVF (39). Our research showed that sperm from bulls fed a diet containing FO and VE reduced the oxidative stress of embryo medium and up-regulated the expression *CAT* and *SOD* genes in blastocysts. In addition, this sperm led to significantly increased blastocyst expression of *CDX2* and *Nanog* genes, which are important for normal embryonic development (40).

It is reported that lanosterol could affect oocyte maturation and subsequent embryonic development (41). Our results showed that a diet supplemented with FO and VE may promote the sperm fertilization rate through increased lanosterol levels, supporting previous findings that supplementation with 50 µM lanosterol during *in vitro* maturation improved preimplantation development of embryos by elevating lipid levels (42). A lack of riboflavin in the diet was reported to reduce murine sperm motility, morphology, and bioenergetic metabolism (43). Riboflavin has antioxidant properties while protecting cells from oxidative stress (44, 45). Similarly, protocatechuic acid can positively affect the reproductive function of male rats through anti-inflammatory and antioxidant effects (46–48). In the current study, we found that dietary FO and VE may enhance bull semen quality by increasing the levels of riboflavin and protocatechuic acid.

Lactate and palmitic acid provide an important energy source for sperm (49, 50). Thus, when high energy is required, sperm efficiently metabolize glycolysable substrates to yield ATP (51). The present study has shown that dietary FO and VE increased the content of lactate and palmitic acid, suggesting the sperm may utilize anaerobic glycolysis more efficiently (52). The current study also found that dietary FO and VE increased aconitase levels in sperm. Studies have shown that mitochondrial aconitase is an important regulator of the TCA cycle in asthenozoospermia, and aconitase was reduced in asthenozoospermia (53). Therefore, these results indicate that high expression of aconitase may promote the TCA cycle and improve sperm motility.

Our results showed that dietary FO and VE increased the expression of lipase A, lysosomal acid type (*LIPA*) and *ABAT* genes in sperm. It has been reported that *LIPA* and *ABAT* are involved in the TCA cycle and maintain mitochondrial membrane function (54, 55). As a key lipid metabolism enzyme, *HADHA* is associated with increased hepatic lipid accumulation when *HADHA* is inhibited, whereas lipid droplet formation is reduced when *HADHA* is overexpressed (56). Enoyl-CoA hydratase and 3-hydroxyacyl CoA dehydrogenase (*EHHADH*) is a part of the fatty acid β-oxidation pathway (57). Our results showed that a diet supplemented with FOVE increased *EHHADH* and *HADHA* gene expression, indicating that sperm lipid metabolism may be regulated through the fatty acid pathway. Aldehyde oxidase 1 (*AOX1*) induces antioxidant defense pathways and increases H_2_O_2_ levels, and subsequently promoting the production of antioxidant enzymes (58). The elevated expression of *AOX1* genes in this study shows that the antioxidant capacity of sperm was increased.

Finally, we performed a combined analysis of metabolomic and transcriptomic data to identify the pathways by which dietary FO and VE affect sperm. We found that propanoate metabolism played an important role in improving sperm motility. Propionate metabolism is downstream of lipid metabolism and contributes to the tricarboxylic acid cycle and provides energy (59). A previous study showed that propanoate metabolic pathways play an important role in sperm cryopreservation (60). In addition, sperm must reserve sufficient energy to sustain motility, capacitation, and fertilization (61). Therefore, dietary FO and VE may affect sperm motility and subsequent embryonic developmental through the propionate pathway.

**Figure 8:**
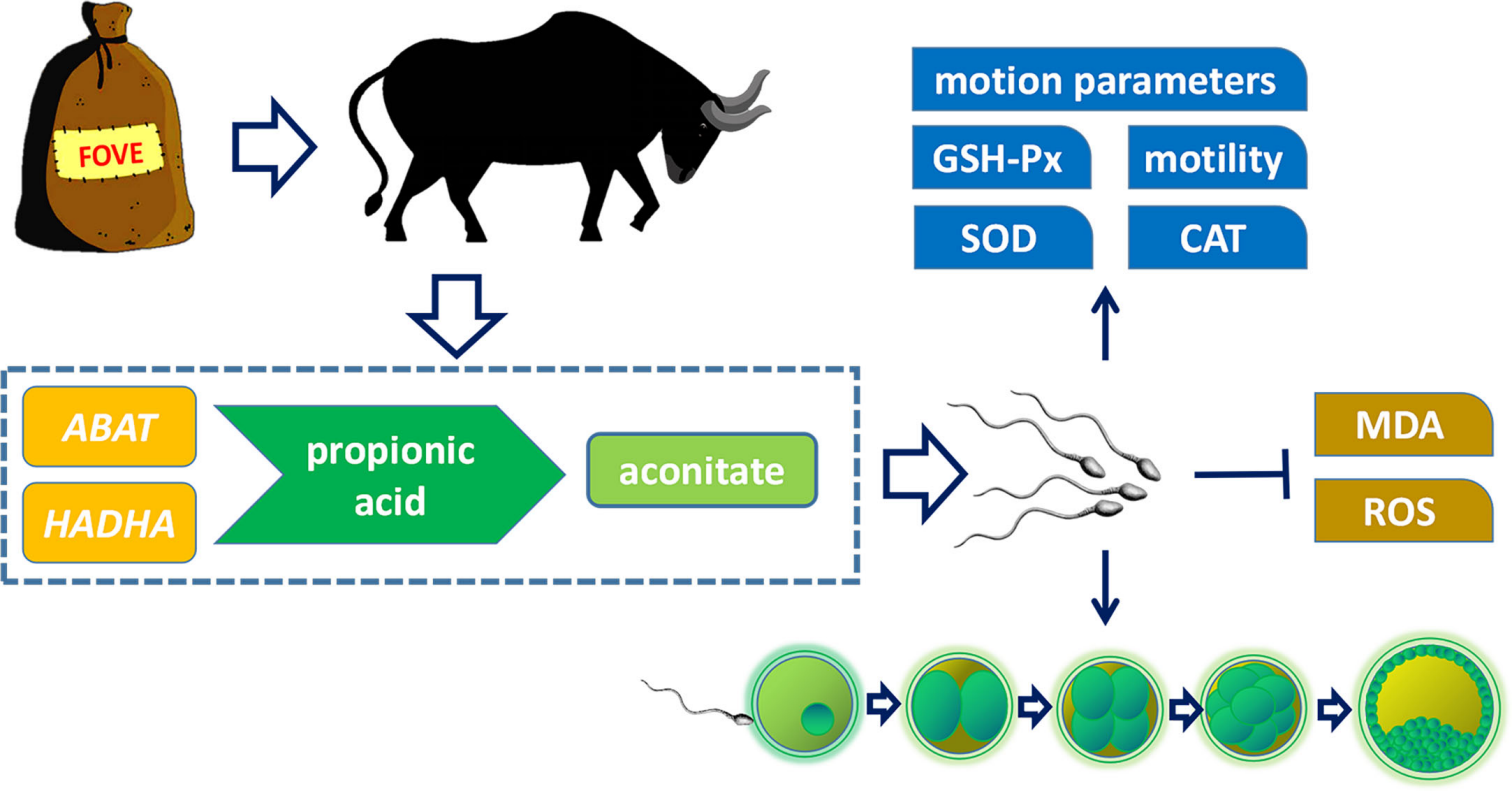
Effects of dietary supplementation of FO and VE on semen quality of bulls. The combination of FO and VE in diet can improve frozen-thawed sperm motility, motion parameters, antioxidant capacity and *in vitro* fertilization ability, which may be caused by the regulation of *ABAT* and *HADHA* genes and aconitate content in propionic acid metabolism pathway.

Based on transcriptome and metabolome analysis, as shown in **Figure 8**, we found that sperm from bulls fed a diet with FO and VE may regulate expression of the *ABAT* and *HADHA* genes in the propionic acid metabolism pathway to increase aconitate, thereby improving semen quality and subsequent embryo development. However, Due to the small number of bulls available for study under actual conditions of the experiment, as a large livestock animal, we cannot obtain a large number of experimental animals to carry out the experiment. In addition, an inherent limitation of this analysis was the in silico platform with results only based on bioinformation instead of direct investigation of biological mechanisms. Therefore, more method validation should be used to validate our hypothesis.

## 5 Conclusions

In conclusion, combined supplementation of FO and VE in bull diets could improve frozen-thawed sperm motion parameters, IVF rate and subsequent embryonic development by enhancing antioxidant capacity, which may be related to increased aconitic acid *via* the *ABAT* and *AHDHA* genes involved in the propionic acid metabolism pathway.

## Data availability statement

The original contributions presented in the study are included in the article/Supplementary Material. Further inquiries can be directed to the corresponding authors.

## Ethics statement

The animal study was reviewed and approved by Jilin Agricultural University in P.R. China and approved by the Experimental Animal Welfare and Ethics Committee of Jilin Agricultural University. Written informed consent was obtained from the owners for the participation of their animals in this study.

## Author contributions

CY wrote the paper. KZ and XM performed the experiments. ZW and JZ did the statistical analysis. HL collected the samples and data. WL and JW edited and reviewed the paper. All authors contributed to the article and approved the submitted version.
